# Value of Hematological and Coagulation Parameters as Prognostic Factors in Acute Coronary Syndromes

**DOI:** 10.3390/diagnostics11050850

**Published:** 2021-05-09

**Authors:** Elena Emilia Babes, Dana Carmen Zaha, Delia Mirela Tit, Aurelia Cristina Nechifor, Simona Bungau, Felicia Liana Andronie-Cioara, Tapan Behl, Manuela Stoicescu, Mihai Alexandru Munteanu, Marius Rus, Mirela Marioara Toma, Ciprian Brisc

**Affiliations:** 1 Department of Medical Disciplines, Faculty of Medicine and Pharmacy, University of Oradea, 410073 Oradea, Romania; babes.emilia@gmail.com (E.E.B.); manuela_stoicescu@yahoo.com (M.S.); mihaimunteanual@yahoo.com (M.A.M.); rusmariusr@yahoo.com (M.R.); brisciprian@gmail.com (C.B.); 2 Clinical Emergency Hospital of Oradea, 410169 Oradea, Romania; danaczaha@gmail.com; 3 Department of Preclinical Disciplines, Faculty of Medicine and Pharmacy of Oradea, University of Oradea, 410073 Oradea, Romania; 4 Department of Pharmacy, Faculty of Medicine and Pharmacy, University of Oradea, 410028 Oradea, Romania; mirela_tit@yahoo.com (D.M.T.); mire.toma@yahoo.com (M.M.T.); 5 Doctoral School of Biomedical Sciences, University of Oradea, 410087 Oradea, Romania; 6 Analytical Chemistry and Environmental Engineering Department, Polytechnic University of Bucharest, 011061 Bucharest, Romania; aureliacristinanechifor@gmail.com; 7 Department of Psycho-Neuroscience and Recovery, Faculty of Medicine and Pharmacy, University of Oradea, 410073 Oradea, Romania; 8 Department of Pharmacology, Chitkara College of Pharmacy, Chitkara University, Punjab 140401, India; tapanbehl31@gmail.com

**Keywords:** acute coronary syndromes, hematological parameters, prognostic factors, coagulation parameters, adverse events

## Abstract

The values of hematological and coagulation biomarkers were evaluated as predictors of in hospital mortality and complications, in patients with acute coronary syndromes (ACS). This retrospective observational study enrolled 936 ACS subjects admitted to the Clinical Emergency Hospital of Oradea, Romania, between January–December 2019. Hematological and coagulation parameters were obtained at admission. During hospitalization, the following adverse events were recorded: death, ventricular rhythm disturbances, atrial fibrillation, heart failure, re-infarction, and stroke. Accuracy of hematological and coagulation parameters as predictors of adverse outcome were also evaluated. The diagnosis was unstable angina in 442 patients (47.22%), non-ST-elevation myocardial infarction (NSTEMI) in 113 patients (12.1%) and ST-elevation myocardial infarction (STEMI) in 381 patients (40.70%); 87 patients (9.29%) died during hospitalization and 193 (20.7%) developed complications. Predictors for in hospital mortality were as follows: red cell distribution width (RDW) (AUC 0.691, *p* < 0.0001), white blood cells (WBC) (AUC 0.684, *p* < 0.0001), neutrophils (NEU) (AUC 0.684, *p* < 0.0001), and prothrombin time (PT) (AUC 0.765, *p* < 0.0001). WBC (AUC 0.659, *p* < 0.0001), NEU (AUC 0.664, *p* < 0.0001), RDW (AUC 0.669, *p* < 0.0001), and PT (AUC 0.669, 95% CI 0.622–0.714, *p* < 0.0001) also had accuracy for complications prediction. RDW had a good ability to predict heart failure in NSTEMI patients (AUC 0.832, *p* < 0.0001). An acceptable ability to predict ventricular rhythm disturbances occurrence had WBC (AUC 0.758, *p* < 0.0001) and NEU (AUC 0.772, *p* < 0.0001). Hematological and coagulation parameters can help in risk stratification of ACS patients. RDW, WBC, NEU, and PT were able to predict mortality and in-hospital complications in ACS patients. RDW has a good accuracy in predicting complications and heart failure in NSTEMI patients. WBC and NEU are good predictors for ventricular rhythm disturbances.

## 1. Introduction

Coronary artery disease (CAD) is the leading cause of death worldwide and is most commonly the result of atherosclerosis. Atherosclerosis is a systemic inflammatory disease, and inflammation plays an important role in the pathophysiology of acute coronary syndromes (ACS). There is a persistent low-grade inflammation that contributes to the initiation and progression of the atherosclerotic process. Atherosclerosis plaques may become unstable leading to thrombosis and development of ACS. Acute coronary syndrome (ACS), including unstable angina (UA), non-ST-segment elevation myocardial infarction (NSTEMI), and ST-segment elevation myocardial infarction (STEMI), is one of the most frequent reasons for hospital admission [1].

Despite the use of guideline recommended therapies including early percutaneous coronary intervention (PCI) the prognosis in ACS is still poor and therefore early risk stratification is needed [2,3].

The inflammation associated with ACS leads to increase research in the field of new inflammatory biomarkers that are useful in the diagnosis and risk stratification of patients. In acute myocardial infarction (MI) there is an increased myeloid activity [4], and the inflammatory process correlated with ACS leads to elevated concentrations of erythropoietin and the presence of hematological markers in the peripheral blood. Many hematological parameters have become increasingly studied as indicators of diagnosis and prognosis in ACS. These hematological indices are widely and easily available in daily clinical practice, are relatively inexpensive and their diagnostic and prognostic value was studied in many diseases including coronary artery disease [4]. Identification of patients at high risk will allow a careful monitoring and intensive treatment to prevent complication and reduce mortality.

In recent years, strong interest was present regarding these parameters, many studies proved their prognostic value for mortality, major cardiovascular events, rhythm disturbances, and stent thrombosis in ACS, while in other studies the results are contradicting their outcome prediction potential. Further studies on these hematological biomarkers are necessary. For example, white blood cell count (WBC) was found to be an important predictor for death in the first 30 days to 6 months following acute MI [5]. Neutrophils to lymphocyte ratio (NLR) is correlated with higher in-hospital mortality in patients with acute MI [6] and a value >4.9 can predict stent thrombosis and increased mortality in patients with acute MI and interventional therapy [4]. Lymphocytes (LYM) are acting as modulators of the inflammation process and a low lymphocyte count is indicator of poor prognostic in patients with acute MI [7]. An elevated mean platelet volume (MPV) was found to be an important predictor of impaired reperfusion and increased six-month mortality in patients with acute MI treated with PCI therapy [4]. Red cell distribution width (RDW) is a predictor of mortality after acute MI in a prospective study of Dabbah et al. and is useful for risk stratification of ACS [8]. Coagulation parameters were less studied as prognostic factors in ACS but there is strong connection between coagulation and inflammation in the pathogenesis of ACS. It seems that prolonged prothrombin time is correlated to mortality in ACS patients [9]. Already available hematological and coagulation parameters can improve risk prediction of death and adverse outcome in ACS patients. More accurate identification of patients at high risk will allow improved treatment and follow-up of those who will benefit the most. Previous studies have mainly focused in predicting long term mortality and complications in ACS patients. However, little is known about the role of these hematological parameters in predicting in-hospital and short-term outcome of patients with ACS.

The aim of this study was to evaluate the value of hematological and coagulation biomarkers as predictors of in-hospital mortality and complications in patients admitted for ACS in a north-western hospital in Romania. The data obtained through this exhaustive research (almost 1000 patients, during 2019) provide valuable information to clinicians in order to better conduct the risk stratification of patients with ACS.

## 2. Materials and Methods

### 2.1. Study Design

A retrospective observational study was performed on 936 patients consecutively admitted with the diagnosis of ACS in the Cardiology Department of the Clinical County Emergency Hospital of Oradea, Romania. Inclusion criteria was the diagnosis of ACS.

**Figure 1:**
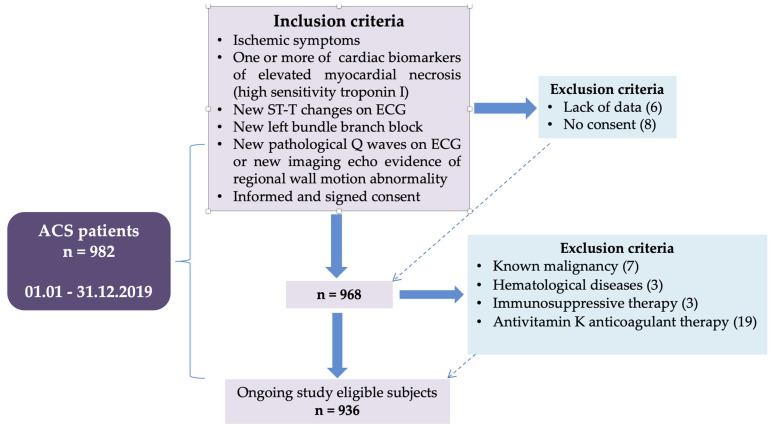
Flow chart presenting the inclusion-exclusion criteria of the patients.

The selection and inclusion-exclusion criteria of the patients are presented in the flow chart (Figure 1).

All the patients had CBC blood sample analysis performed at admission. Coagulation tests were missing in 43 patients. Ejection fraction was not registered in 21 patients. Patients with missing data were mainly in the group with unstable angina.

The following adverse events were recorded during hospitalization period: death and other major complications like ventricular rhythm disturbances, development of acute MI for patients with unstable angina (UA) or re infarction, heart failure, new onset atrial fibrillation, and stroke. Hematological and coagulation parameter obtained at admission were compared between groups of patients with/without adverse events, and those parameters that differed significantly between groups were evaluated regarding their predictive accuracy.

### 2.2. Methodology

ACS consisted of: UA, NSTEMI, and STEMI. The diagnosis of UA was defined in the presence of acute chest discomfort described as chest pain or chest pain equivalent symptoms (dyspnea) and ECG changes indicating myocardial ischemia: persistent or transient ST depression, T wave inversion, flat T waves, pseudonormalization of T waves, without evidence of cardiomyocyte necrosis, respectively with normal high-sensitivity cardiac troponin I value [10]. NSTEMI was diagnosed if acute chest discomfort or ECG changes indicating myocardial ischemia were associated with elevation of cardiac markers of myocardial necrosis, respectively high sensitivity troponin I [10]. STEMI was defined if acute chest discomfort was associated with persistent ST elevation in at least two contiguous leads, with an amplitude measured at J point of ≥2.5 mm in men <40 years, ≥2 mm in men ≥40 years, or ≥1.5 mm in women in leads V2-V3 and/or ≥1 mm in the other leads (in the absence of left ventricular hypertrophy or left bundle branch block) [2].

Data were obtained from patient’s medical records and from hospital informatic system. Demographic data, medical history, clinical and paraclinical data, and risk factors were recorded.

Hematological parameters were assessed at admission by using CELL-DYN Ruby System (Abbot, USA), an automated hematology analyzer that perform cell by cell analysis from a single sample dilution to count and differentiate white blood cells using Multi-Angle Polarized Scatter Separation (MAPSS) technology. The device also uses optical laser light scatter analysis, without the need to reflex to another test mode, to provide both the red blood cell and platelet counts. Coagulation tests performed at admission were registered: prothrombin time (PT) and activated partial thromboplastin time (APTT). For this purpose, it was used ACL TOP Family 500 CTS (Werfen, Barcelona).

Atrial fibrillation was defined as heart rhythm with no discernible P waves and irregular RR intervals lasting at least 30 s [11]. Ventricular arrhythmias were defined as ventricular tachycardia or ventricular fibrillation that were recorded during ECG monitoring or on a 12 leads ECG.

Heart failure was appreciated by corroborating clinical signs with echocardiographic data. Left ventricular ejection fraction was appreciated with Simpson s method, recommended by the American Society of Echocardiography and the European Association of Cardiovascular Imaging [12].

Stroke was diagnosed based on neurological evaluation and cerebral computed tomography scanning. Re-infarction was defined as an acute MI that appeared after the incident Mi and was manifest as reappearance of ischemic symptoms associated with new ST elevation or new pathognomonic Q waves in at least two contiguous leads and confirmed by high sensitivity troponin assays (two samples at 3–6 h apart). If the initial value was elevated but stable or decreasing, the second troponin value required a >20% increasing, if the initial value was normal than criteria for new acute MI apply [13].

### 2.3. Statistical Analysis

Data were analyzed using SPSS statistical package version 25 and MedCalc statistical software. Results are presented as mean ± SD for continuous variable and frequencies and percentage for categorical variables. Intergroup comparison was made using independent sample *t*-test for continuous variables and Kruskal–Wallis test for categorical variable. A value of *p* < 0.05 was considered statistically significant.

For those hematological and coagulation parameters that were significantly different between various groups a ROC curve analysis was performed with determination of AUC (area under curve) to evaluate the accuracy or the performance of these parameters in predicting mortality or adverse outcome. Optimal cutoff value with highest Youden index was determined for each parameter

Demographic data (age, sex), clinical (diagnosis, comorbid conditions) and paraclinical variables (LVEF, creatinine, hematological, and coagulation parameters) that were statistically significant different between various groups were included in a multiple regression analysis in order to evaluate the independent predictive value for each parameter. In order to evaluate the independent predictive value for mortality, the multivariate Cox regression analysis was performed.

## 3. Results

The diagnosis was unstable angina in 442 patients (47.22%), NSTEMI in 113 patients (12.1%), and STEMI in 381 patients (40.70%). Mean age was 65.64 ± 11.794 years and 64.1% were male. Coronary angiography was performed in 720 patients (76.9%) and 490 (52.4%) patients with suitable anatomy were treated with interventional revascularization. The rest of the patients were sent to coronary artery bypass (79 patients, 8.4%) or had nonsignificant (<50%) coronary artery stenosis. A number of 625 patients (66.8%) were hypertensive, 424 patients were with dyslipidemia (45.3%), 294 patients (31.4%) had diabetes, and 224 patients were smokers (23.9%). Duration of hospitalization was 5.6 ± 4.92 days.

### 3.1. Hematological and Coagulation Parameters as Prognostic Factors for Mortality

The mortality rate during hospitalization was 9.29% (87 patients). Comparison of baseline characteristics: clinical and paraclinical features in patients discharged alive and deceased are represented in Table 1.

The following hematological parameters had discriminative power in predicting mortality: hemoglobin (HGB) (AUC 0.664, 95% CI 0.633–0.694, *p* < 0.001), WBC (AUC 0.684, 95% CI 0.650–0.718, *p* < 0.001), and NEU (AUC 0.694, 95% CI 0.660–0.727, *p* < 0.001). A cut off value of 12.5 g/dL for HGB has a sensitivity of 49.4% and a specificity of 79.6% in identifying patients at risk of death. A cut off value of 15.36 × 10³/μL for WBC can identify with a sensitivity of 43.2% and a specificity of 88.9% a higher risk of mortality. NEU value higher than 8.3 × 10³/μL has a sensitivity of 64% and a specificity of 69.2% in predicting mortality. NLR had an AUC of 0.626, 95% CI 0.594–0.656, (*p* = 0.008), and a value >5.116 indicates with a sensitivity of 48.28% and a specificity of 81.23% mortality risk. RDW was also able to predict mortality with an AUC 0.691, 95% CI 0.656–0.724, (*p* < 0.001) and with a cut off value of 12.48% having a sensitivity of 67.1% and a specificity of 66.7% for detecting patients with high risk of mortality.

**Figure 2:**
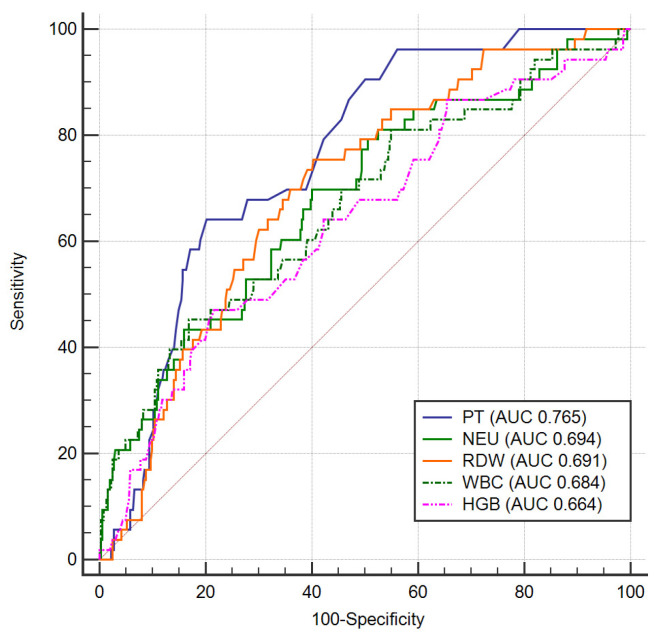
ROC curves for mortality prediction in ACS. Legend: PT—prothrombin time; NEU—neutrophils; RDW—red cell distribution width; WBC—white blood cells; HGB—hemoglobin; ACS —acute coronary syndrome.

Better discriminative power was registered for PT (AUC 0.765, 95% CI 0.722–0.804, *p* < 0.001). A cut off value of 14.95 s for PT has a sensitivity of 64.8% and a specificity of 80.1% in detecting patients with higher risk of death. The comparative value of the parameters as predictors of mortality in ACS patients is revealed in Figure 2, by comparing ROC curves.

In multivariate Cox regression analysis WBC remained independent predictors of mortality (HR = 1.094, 95% CI: 1.025–1.167; *p* = 0.007). Other independent predictors of death were: LVEF (HR = 0.892, 95% CI: 0.859–0.926; *p* < 0.001), creatinine (HR = 1.74, 95% CI: 1.22–2.50; *p* = 0.002), diagnosis of STEMI (HR = 4.96, 95% CI 1.95–12.64, *p* = 0.001), and age (HR = 1.06, 95% CI 1.028–1.098, *p* < 0.001).

For the patients with diagnosis of UA, PLR was significantly higher in the mortality group (171.64 ± 10.57 vs. 115.54 ± 56.66, *p* = 0.04) and the discriminative power of this parameter was good (AUC 0.891, 95% CI 0.850–0.923, *p* < 0.001), with a cut off value of 163.625 (sensitivity 100% and 87.9% specificity) indicating a group of patients at high risk of death, but there only three patients with UA in the mortality group.

Thirteen patients with diagnosis of NSTEMI died during hospitalization period. In these patients, WBC were significantly higher (Table 2) with a fair discriminative ability in predicting death (AUC 0.780, 95% CI 0.675–0.864, *p* = 0.016). A cut off value of 15.36 × 10³/μL can identify with a sensitivity of 63.6% and a specificity of 87.3% patients with high risk of mortality. RDW was also significantly higher in the mortality group and had an acceptable discrimination power (AUC of 0.778, 95% CI 0.671–0.863, *p* < 0.001). A value of more than 12.73% can identify with a sensitivity of 72.7% and a specificity of 68.1% patients with increased risk of death.

A number of 71 deaths were registered among patients with STEMI. Coagulation parameter PT was significantly higher in the mortality group (Table 3). This parameter had a good discriminative ability (AUC 0.817, 95% CI 0.764–0.862, *p* < 0.001), and a cut off value of 14.9 s can indicate with a sensitivity of 62.22% and a specificity of 90.32% a higher risk for death. RDW was significantly higher in the mortality group in STEMI patients with a fair discriminative power (AUC 0.702, 0.651–0.749, *p* < 0.001). A cut off value of 12.51% has 66.7% sensitivity and 72.1% specificity in detecting high risk of mortality.

From ACS patients treated with PCI and stenting a number of 18 patients died. Deceased patients treated with interventional revascularization had significantly higher NLR 6.38 ± 7.02 vs. 3.66 ± 3.72 (*p* = 0.004) and PLR 169.23 ± 163.28 vs. 122.388 ± 71.15 (*p* = 0.02). NLR (AUC 0.540, 95% CI 0.494–0.585, *p* = 0.66) and PLR (AUC 0.530, 95% CI 0.480–0.579, *p* = 0.78) failed in predicting death in patients with PCI.

### 3.2. Hematological and Coagulation Parameters as Prognostic Factors for Complications

A number of 193 patients (20.7%) developed complications during hospitalization. Atrial fibrillation occurred in 35 patients (3.7%), ventricular rhythm disturbances appeared in 56 patients (6%), 116 patients developed heart failure (12.4%), 15 patients (1.6%) had a new myocardial ischemic event (MI or re infarction) and 7 patients (0.7%) developed ischemic stroke. A number of 26 patients (2.77%) developed more than one complication. Comparative clinical and paraclinical parameters in patients with and without in-hospital complications are shown in Table 4.

WBC (AUC 0.659, 95% CI 0.624–0.693, *p* < 0.001), NEU (AUC 0.664, 95% CI 0.629–0.698, *p* < 0.001), RDW (AUC 0.669, 95% CI 0.634–0.703, *p* < 0.001) were found to have ability for complications prediction. NLR had very poor discriminative value for complications (AUC 0.586, 0.554–0.619, *p* = 0.0008). Cut off values of 13.92 × 10³/μL for leucocyte (sensitivity 42.3%, specificity 84.6%) can identify patients predisposed to complications. For NEU a cut off value of 6.82 × 10³/ μL can identify complication predisposed patients with a sensitivity of 67.5% and a specificity of 59.1%. Moreover, WBC persists as independent predictor for complications in ACS patients after multiple regression analysis (HR = 1.345, 95% CI 1.093–1.655, *p* = 0.005).

RDW had a cut off value of 12.53% (sensitivity 57.8%, specificity 71.6%) and NLR had a cut of value of 4.39 (sensitivity 44.7%, specificity 77.6%) for complication prediction. In addition, RDW was an independent predictor of complications in a multivariate regression analysis (HR = 1.164, 95% CI 1.020–1.330, *p* = 0.025). Other independent predictors were as follows: age (HR = 1.031, 955 CI 1.009–1.054, *p* = 0.006), LVEF (HR = 0.940, 95% CI 0.917–0.964, *p* < 0.001), creatinine (HR = 1.54, 95% CI 1.119–2.118, *p* < 0.001), diagnosis of STEMI (HR = 1.797, 95% CI 1.025–3.149, *p* = 0.04).

**Figure 3:**
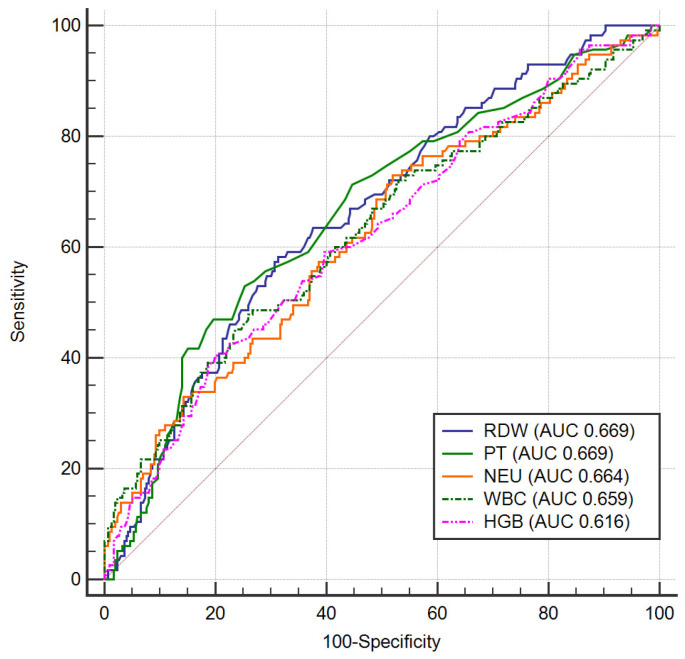
ROC curves for complications prediction in ACS. Legend: RDW—red cell distribution width; PT—prothrombin time; NEU—neutrophils; WBC—white blood cells; HGB—hemoglobin; ACS—acute coronary syndrome.

HGB had also an ability in predicting complications (AUC 0.616, 95% CI 0.584–0.648, *p* < 0.001) and a value lower than 13.64 g/dL has a sensitivity of 59.6% and a specificity of 60% for complication detection. Another parameter that can predict complications was found to be PT (AUC 0.669, 95% CI 0.622–0.714, *p* < 0.001) with a cut off value of 14.4 s (sensitivity 53.4%, specificity of 75.3%). The best hematological parameters predictors of complications are revealed in Figure 3 by ROC curve comparison, there is no statistically significant differences between parameters regarding prediction accuracy.

A number of 43 patients with UA developed complications. In these patients, RDW was significantly increased (12.96 ± 1.15% vs. 12.42 ± 1.45%, *p* = 0.02). The AUC was 0.669, 95% CI 0.614–0.722, (*p* = 0.0016) and RDW value of more than 13.14% can identify with a sensitivity of 46.3% and a specificity of 82.37% complications risk.

There were 30 patients among those with diagnosis of NSTEMI that developed complications. NEU and WBC were significantly higher in patients with NSTEMI (Table 5) who developed complications and were found to have an acceptable ability to diagnose patients with complication occurrence (AUC 0.696, 95% CI 0.585–0.793, *p* = 0.0052 for NEU, and 0.709 for WBC, 95% CI 0.598–0.804, *p* = 0.0057). A cut off value of 6.13 × 10³/μL for NEU can identify with a sensitivity of 85% and a specificity of 53.2% the patients who will develop complications. WBC had a cut off value off 14.38 × 10³/μL that can identify with a sensitivity of 55% and a specificity of 83.9% the patients predisposed to complications.

RDW had a fair discriminative value for complications in patients with NSTEMI (AUC 0.767, 95% CI 0.659–0.854, *p* = 0.001) with a cut off value of 14.04% (sensitivity 55%, specificity 93.3%).

HGB was significantly lower in the group of patients with complicated STEMI (Table 6) and had an AUC 0.670, 95% CI 0.620–0.717, (*p* < 0.001). A value lower than 13.4 g/dL had a sensitivity of 56.7% and a specificity of 72.4% for complication prediction. Acceptable discriminative power for complications occurrence was found also for PT (AUC 0.705, 95% CI 0.646–0.760, *p* = 0.005), with a cut of value of 14.7 s (sensitivity 45.4%, specificity 77.9%).

RDW is also able to detect complications prone STEMI patients (AUC 0.673, 95% CI 0.621–0.722, *p* < 0.001) with a cut off value 12.51% (sensitivity 57.5%, specificity 75.4%).

WBC, NEU, and NLR were significantly higher in STEMI patients that developed complications. NLR had an AUC of 0.599, 95% CI 0.499–0.601, (*p* = 0.14) and fail to identify patients at risk of complications. WBC (AUC 0.590, 95% CI 0.546–0.650, *p* = 0.005) with a value higher than 14.6 × 10³/μL is correlated with risk of complications with a sensitivity of 45.4% and a specificity of 77.9%. NEU (AUC 0.590. 95% CI 0.538–0.642, *p* = 0.01) with a value higher than 13.56 × 10³/μL can be correlated with complications with a sensitivity of 33% and a specificity of 88.8%. The accuracy of WBC and NEU for complication prediction in STEMI patients was poor.

PT was significantly increased in patients with complicated STEMI, and had an acceptable discriminative power (AUC 0.705, 95% CI 0.646–0.760, *p* < 0.001). A value over 14.7 s has a sensitivity of 42.17% and a specificity of 89.39% for predicting complications. As well, PT persisted as independent predictor of complications in STEMI patients after multiple regression analysis (HR = 1.399, 95% CI 1.083–1.808, *p* = 0.01). Other independent predictors were LVEF (HR = 0.927, 95% CI 0.895–0.960, *p* < 0.001), age (HR = 1.033, 95% CI 1.001–1.066, *p* = 0.04), and creatinine (HR = 3.497, 95% CI 1.439–8.5).

A number of 86 ACS patients treated with interventional revascularization therapy developed in-hospital complications. RDW was significantly higher in stented patients that developed complications (Table 7). The AUC was 0.646 (95% CI 0.597–0.692, *p* < 0.0001) with a cut off value of 11.92% (sensitivity 76.8%, specificity 48.5%).

PT was also significantly higher in patients treated with PCI that developed complications and had an AUC 0.638 (95% CI 0.573–0.699, *p* = 0.001). A cut of value of 14.7 s can discriminate with a sensitivity of 35.7% and a specificity of 89.6% patients treated with interventional revascularization and stenting that are at risk for complications.

WBC (AUC 0.598, 95% CI 0.548–0.645, *p* = 0.017), NEU (0.590, 95% CI 0.540–0638, *p* = 0.022) and HGB (AUC 0.583, 95% CI 0.538–0.627, *p* = 0.016) were significantly higher in stented patients who developed complications but had a poor discriminative power for complications in patients treated with interventional revascularization therapy. NLR (0.525, 95% CI 0.479–0.570, *p* = 0.51) and PLR (0.501, 95% CI 0.452–0.551, *p* = 0.97) were higher in the complications group but fail to identify patients at risk for complications.

### 3.3. Hematological and Coagulation Parameters as Prognostic Factors for Ventricular Rhythm Disturbances

HGB level was significantly lower (*p* < 0.001), PLR (*p* = 0.01), NLR (*p* < 0.001), NEU (*p* < 0.001), WBC (*p* < 0.001), and RDW (*p* < 0.001) were significantly higher in the group of patients with ACS that developed ventricular rhythm disturbances (Table 8).

An acceptable ability to predict ventricular rhythm disturbances occurrence in ACS patients had WBC (AUC 0.758, 95% CI 0.726–0.788, *p* < 0.001) and NEU (AUC 0.772, 95% CI 0.741–0.802, *p* < 0.001). A cut off value for WBC of 9.94 × 10³/μL can indicate risk of ventricular arrhythmias with a sensitivity of 85.7% and a specificity of 54.6%. NEU with a value over 7.784 × 10³/μL is associated with ventricular rhythm disturbances with a sensitivity of 79.59% and a specificity of 64.95%.

NLR (AUC 0.675, 95% CI 0.644–0.706, *p* < 0.001) and HGB (AUC 0.623, 95% CI 0.591–0.654, *p* = 0.002) had also discriminative power to detect ventricular rhythm disturbances. The cut off value of 4.25 for NLR is correlated with ventricular rhythm disturbances with a sensitivity of 60.7% and a specificity of 73.6%. A HGB level lower than 12.8 g/dL can detect with a sensitivity of 48.2% and a specificity of 74.1% the risk of ventricular rhythm disturbances in ACS patients.

RDW had also an AUC 0.691, 95% CI 0.656–0.724, *p* < 0.001 and a cut off value >12.48% can identify ventricular arrhythmia risk with a sensitivity of 66.7% and a specificity of 65.4%. Also, RDW was an independent predictor of ventricular arrhythmias in ACS patients even after multivariate regression analysis (HR = 1.24, 95% CI 1.026–1.505, *p* = 0.026) LVEF (HR = 0.939, 95% CI 0.904–0.975, *p* = 0.001).

PLR had an AUC of 0.573, (95% CI 0.537–0.609, *p* = 0.14) and failed regarding ability to identify patients at risk for ventricular rhythm disturbances. The best accuracy in predicting ventricular rhythm disturbances in ACS patients was found for WBC and NEU.

NLR was significantly increased in patients with UA that developed ventricular arrhythmias (4.71 ± 0.44 vs. 2.29 ± 2.84, *p* = 0.04) but only 2 patients developed severe ventricular arrhythmias, so the parameter was not able to discriminate the risk of ventricular rhythm disturbances (AUC was 0.517, 95% CI 0.468–0.565, *p* = 0.96).

In STEMI group, there were 49 patients with severe ventricular arrhythmias. Hematological and coagulation parameters in STEMI patients are revealed in Table 9.

The following parameters were found to have an acceptable ability in predicting ventricular arrhythmias in STEMI patients: RDW (AUC 0.724, 0.764–0.770, *p* < 0.001) and PT (AUC 0.733, 95% 0.675–0.785, *p* < 0.001). Cut off values were 11.92% for RDW (sensitivity 93.02%, specificity 43.65%) and 13.3 s for PT (sensitivity 97.3%, specificity 41.33%). RDW remained independent predictor of ventricular rhythm disturbances in STEMI patients after multivariate analysis (HR = 1.304, 95% CI 1.069–1.591, *p* = 0.009).

WBC, NEU, and HGB can discriminate STEMI patients with ventricular arrhythmias risk but with a lower accuracy. AUC was 0.634 for leukocyte, 95% CI 0.581–0.684, (*p* = 0.0048), 0.647 for NEU, 95% CI 0.595–0.697, (*p* = 0.001) and respectively 0.657 for HGB, 95% CI 0.607–0.704, (*p* = 0.0003). Cut off values were 13.48 × 10³/μL for leucocyte (sensitivity 52.27%, specificity 63.26%), 7.744 × 10³/μL for NEU (sensitivity 79.55%, specificity 44.90%), 12.9 g/dL for HGB (sensitivity 51% and specificity 75.9%).

A number of 24 patients with interventional revascularization therapy from the ACS group that developed ventricular arrhythmias had significantly increased WBC, NEU, and NLR (Table 10). WBC and NEU had the best discriminative value with an AUC of 0.776, 95% CI 0.732–0.815, (*p* < 0.001) and respectively 0.772, 95% CI 0.728–0.811, (*p* < 0.001). The cut off values were 14.6 × 10³/μL for WBC (sensitivity 63%, specificity 84.5%) and 7.763 × 10 ³/μL for NEU (sensitivity 86.4%, specificity 80.7%). NLR was a poor predictor of ventricular rhythm disturbances (AUC 0.650, 95% CI 0.605–9.692, *p* = 0.015), with a cut of value of 3.93 (sensitivity 58.3%, specificity 68%).

### 3.4. Hematological and Coagulation Parameters as Prognostic Factors for Atrial Fibrillation

LR and NLR were significantly higher in the group of patients with ACS that developed atrial fibrillation (176.80 ± 146.22 vs. 130.25 ± 83.53, *p* < 0.001, and respectively 5.27 ± 6.23 vs. 3.74 ± 4.40, *p* = 0.013) but these parameters failed as predictors of atrial fibrillation in the studied group (AUC 0.557, 95% 0.520–0.593, *p* = 0.36 and respectively 0.561, 95% CI 0.528–0.594, *p* = 0.22).

There were 18 patients with STEMI that developed atrial fibrillation. NLR was significantly increased in these patients (8.11 ± 7.49 vs. 5.35 ± 4.93, *p* = 0.025) but it was not a reliable predictor for atrial fibrillation (AUC 0.609, 95% CI 0.557–0.658, *p* = 0.16). PLR was also higher in patients with STEMI who developed atrial fibrillation (236.772 ± 171.135 vs. 140.18 ± 93.86, *p* < 0.001) with AUC 0.660, 95% CI 0.608–0.709, (*p* = 0.06) and no accuracy for prediction of atrial fibrillation.

In ACS patients treated with PCI who developed atrial fibrillation (*n* = 20) NLR was significantly increased 5.92 ± 7.87 vs. 3.68 ± 3.65, (*p* = 0.01) and also PLR was increased 197.74 168.04 vs. 121.10 69.26 (*p* < 0.001). NLR had an AUC 0.502, 95% 0.456–0.547 (*p* = 0.96) and PLR had an AUC 0.615, 95% CI 0.566–0.663, (*p* = 0.18) and were not able to discriminate atrial fibrillation risk in this group of patients.

### 3.5. Hematological and Coagulation Parameters as Prognostic Factors for Heart Failure

HGB level was significantly lower (*p* = 0.006), NEU (*p* < 0.001), WBC (*p* < 0.001), PLR (*p* < 0.001), and NLR (*p* < 0.001) were significantly higher in ACS patients who developed heart failure (Table 11).

NLR had an AUC of 0.602, 95% CI 0.569–0.634, *p* = 0.0014 signifying a poor ability to predict heart failure. A value of more than 4.653 can predict heart failure development with a sensitivity of 46.1% and a specificity of 78.6%.

WBC (AUC 0.637, 95% CI 0.601–0.671, *p* = 0.001), NEU (AUC 0.636, 95% CI 0.601–0.671, *p* < 0.001), and HGB (AUC 0.616, 95% CI 0.584–0.647, *p* = 0.0001) have discriminative power for heart failure detection. A cut off value of 13.92 × 10³/μL for WBC has sensitivity of 43.9% and a specificity of 82.4%, and a value over 6.845 × 10³/μL for NEU has a sensitivity of 65.7% and a specificity of 56.7% for predicting heart failure development. In a multivariate regression analysis WBC (HR = 1328, 95% CI 1.065–1.656, *p* = 0.012) remained independent predictor of heart failure. Other independent predictors were age (HR = 1.051, 95% CI 1.026–1.077, *p* < 0.001) and creatinine (HR = 1.51, 95% CI 1.132–2.014, *p* = 0.005). A cut off value of 13.6 g/dL for HGB has a sensitivity of 62.93% and a specificity of 59.02% for heart failure prediction.

A number of 16 patients with UA developed heart failure and PDW was significantly increased in this group, 21.13 ± 1.82% vs. 20.07 ± 1.30%, (*p* = 0.005) but failed to discriminate for heart failure development (AUC 0.654. 95% CI 0.598–0.708, *p* = 0.102).

In NSTEMI patients who developed heart failure (*n* = 22), WBC (*p* = 0.005), NEU (*p* = 0.001), PLR (*p* = 0.034), and also RDW (*p* = 0.026) were significantly higher (Table 12).

RDW had a good ability to predict heart failure in NSTEMI patients (AUC 0.832, 95% CI 0.731–0.906, *p* < 0.0001) and a value over 14.04% can identify patients at risk of heart failure with a sensitivity of 68.7% and a specificity of 93.7%. RDW persisted as an independent predictor for heart failure after multiple regression analysis (HR = 6.385, 95% CI 1.928–21.151, *p* = 0.002). NEU and WBC also had an acceptable ability to predict heart failure (AUC 0.709, 95% CI 0.599–0.804, *p* = 0.004 and respectively 0.717, 95% CI 0.607–0.811, *p* = 0.007). NEU of more than 6.13×10 ³/μL had a sensitivity of 87.5% and a specificity of 51.5% for heart failure occurrence; WBC of more than 11.86 × 10 ³/μL are predicting heart failure with a sensitivity of 68.75% and a specificity of 69.7%. PLR (AUC 0.514, 95% CI 0.401–0.626, *p* = 0.88) failed to prognosticate heart failure in NSTEMI patients.

A number of 78 patients with STEMI developed heart failure and increased WBC (*p* = 0.001), NEU (*p* = 0.001), PLR (*p* = 0.024), and NLR (*p* = 0.027) were found in these patients (Table 13). However, WBC (AUC 0.556, 95% CI 0.503–0.609, *p* = 0.19), NEU (AUC 0.545, 95% CI 0.491–0.597, *p* = 0.3), PLR (AUC 0.558, 95% CI 0.504–0.610, *p* = 0.17), and NLR (AUC 0.539, 95% CI 0.487–0.590, *p* = 0.3) did not have enough accuracy for detecting heart failure.

In ACS patients treated with PCI who developed heart failure (*n* = 42), PT (*p* = 0.04), NLR (*p* = 0.02), PLR (*p* = 0.03), and RDW (*p* < 0.001) were significantly increased (Table 14). The only parameter able to predict heart failure was RDW (AUC 0.680, 95% CI 0.632–0.725, *p* = 0.0001) with a cut off value of 12.53% (sensitivity 56.76%, specificity 75.14%). The rest of parameters PT (AUC 0.582, 95% CI 0.517–0.646, *p* = 0.17), NLR (AUC 0.539, 95% CI 0.487–0.590, *p* = 0.34), and PLR (AUC 0.558, 95% CI 0.504–0.610, *p* = 0.17) failed to predict heart failure in patients treated with interventional revascularization therapy.

### 3.6. Hematological and Coagulation Parameters as Prognostic Factors for New Ischemic Event

Only four patients with UA developed during hospitalization a MI. A lower lymphocyte number was found in these patients (1.31 ± 0.66 × 10 ³/μL vs. 2.12 ± 0.81 × 10³/ μL, *p* = 0.045). The AUC was 0.790, 95% CI 0.740–0.834, *p* = 0.016) that denotes an acceptable ability to predict future short term ischemic events with a cut off value of 1.398 (sensitivity 75%, specificity 82.6%).

NLR was increased in UA patients that developed in-hospital MI (9.43 ± 10.52 vs. 2.24 ± 2.61, *p* < 0.001) and has an excellent predictive value for short term ischemic events (AUC 0.888, 95% CI 0.854–0.916, *p* < 0.001). A cut off value of 4.04 has a sensitivity of 75% and a specificity of 87.7%. PLR was higher in patients complicated with MI (181.28 ± 112.58 vs. 115.04 ± 55.21, *p* = 0.02) and has an acceptable ability to predict ischemic events on short term (AUC 0.717, 95% CI 0.663–0.767, *p* = 013). A cut off value of 178.224 has a sensitivity of 50% and a specificity of 90.5%.

A number of six patients with NSTEMI and five patients with STEMI developed re-infarction during hospitalization period but hematological and coagulation parameters were not found to be significantly different in these patients.

In ACS patients treated with PCI that experienced recurrent myocardial ischemic event (*n* = 4), PT (*p* < 0.001), NLR (*p* = 0.001), and PLR (*p* = 0.03) were significantly higher, and lymphocytes (*p* = 0.03) were significantly lower (Table 15). The best discriminative value is present for lymphocytes with an AUC of 0.920, 95% CI 0.889–0.944, *p* < 0.0001 and a cut of value of 1.398 (sensitivity 100%, specificity 81.8%). PLR had a good discriminative ability (AUC = 0.814, 95% CI 0.773–0.851, *p* = 0.01) with a cut off of 197.088 (sensitivity 66.7%, specificity 90.6%). NLR (AUC 0.708, 95% CI 0.665–0.748, *p* = 0.33) failed to predict myocardial ischemia on short term.

### 3.7. Hematological and Coagulation Parameters as Prognostic Factors for Stroke

There were seven patients with ACS that developed ischemic stroke during hospitalization. The studied hematological and coagulation parameters did not differ significantly between patients with and without stroke and were not able to predict this complication in the studied group.

Lymphocyte number were significantly lower in patients with three vessel disease (2.10 ± 1.01 × 10³/μL) compared with one vessel (2.35 ± 1.14 × 10³/μL) (*p* = 0.02). Lymphocyte had a very poor discriminative value with an AUC of 0.570, 95% CI 0.521–0.618, (*p* = 0.012). A value of ≤1.982 × 10³/μL had 55.96% sensitivity and 58.04% specificity to identify a more extensive coronary artery disease.

## 4. Discussion

Several hematological parameters and coagulation parameter PT were prognostic factors for in-hospital outcome in ACS patients. RDW is a routinely reported parameter in a CBC and is a measure of variations in the volume of red blood cells. In our study, RDW was found to have an acceptable ability to predict mortality and complications in ACS patients. The best discriminative ability for mortality risk was found in patients with NSTEMI and STEMI. However, similar results were found in other studies [7,14,15,16,17,18]. An increased admission RDW is correlated with in-hospital and long-term mortality in STEMI patients undergoing primary PCI in a retrospective study performed by Uyarel et al. [14] and RDW is a predictor of mortality and of adverse clinical outcome in patients with acute MI in a prospective study performed by Dabbah et al. [7]. RDW is correlated with increased incidence of future cardiovascular events and mortality in patients with coronary artery disease and had a strong predictive value for mortality and major cardiac events in patients with acute MI [15]. In the study of Sun et al. on 691 patients with STEMI, high RDW levels were associated with all-cause mortality [16], and in the study of Gul at al. on patients with NSTEMI and unstable angina the mortality rate was significantly higher in the group with high RDW [17]. In our study, RDW had a fair discriminative power for detecting specific complications like ventricular rhythm disturbances in STEMI patients and a good ability to predict heart failure in patients with NSTEMI. Similar results were reported by authors in a review of 13 trials where a lower RDW is associated with a lower risk of major cardiac events after an ACS [18].

In other studies [19,20], it seems that a RDW level over 13.9% can predict stent thrombosis with a sensitivity of 57% and a specificity of 52% in patients with STEMI undergoing primary PCI [19]. RDW was also significantly higher in stented patients that developed complications in the present study and a value over 11.92% can predict complications in patients with interventional treatment with a sensitivity of 76.8% and a specificity of 48.5%. Similar results were observed in the study of Chang et al. where a combination of RDW and GRACE score may be valuable for predicting major cardiac events and death in patients with STEMI treated with PCI [20].

Anisocytosis is an important predictor of severity of coronary artery disease in patients with acute MI [6,21]. Inflammation cause changes in erythrocyte maturation with the release in circulation of immature erythrocyte and adrenergic activation in ACS can influence erythropoiesis [6]. An increased RDW leads to a reduced deformability of red blood cells which can impair blood flow in microcirculation diminishing oxygen supply. In the prospective study performed by Lippi et al., RDW at admission combined with other conventional cardiac markers is useful for risk stratification of ACS patients in the emergency department [22].

In the case of patients with cardiovascular disease, the elucidation of the mechanisms by which high RDW values are correlated with the adverse result is not yet fully established, they are being most likely multifactorial. Regarding the adverse outcome after ACS, numerous parameters were observed (i.e., an increase in oxidative stress, inflammatory cytokines, adrenergic activation, and neurohumoral pathways). In addition, there is a possibility that all of the aforementioned mechanisms may be involved in both erythroid cells poiesis and bone marrow response [23].

WBC are involved in the destabilization of atherosclerosis plaque of ACS patients. In the present study, admission WBC and NEU count were correlated with in-hospital mortality in ACS, especially in NSTEMI patients. Other studies found similar results: elevated WBC was a relevant risk factor for death during the first 30 days to 6 months in patients with UA and NSTEMI in the study of Sabatine et al. [5] and elevated WBC correlated with high mortality rates in patients with MI in the study of Barron et al. [24]. Leukocytosis is associated with an increased cardiovascular mortality rate and with adverse clinical outcomes in many other studies [25]. In a retrospective study performed by Cannon et al., WBC >10,000 × 10³/μL was associated with increased 30 days and 10-month mortality [26]. In our study a cut off value of 15.36 × 10³/μL has a 43.2% sensitivity and 88.9% specificity in predicting in-hospital mortality among patients with ACS.

Leukocyte and NEU had an acceptable predictive power for adverse outcome in patients with ACS, especially NSTEMI patients. In patients with STEMI or in patients treated with PCI the discriminative power was lower. However, in another study performed by Chia et al. in patients with STEMI elevated WBC and neutrophils are found to be predictors of adverse cardiac events [27]. In patients with ACS undergoing PCI leukocytosis was able to predict short-term mortality in a retrospective study performed by Anjarwani et al. [28].

WBC and NEU were found to have an acceptable discriminative power for predicting ventricular rhythm disturbances in ACS patients and in patients treated with PCI. Rahimi et al. in a multicenter study revealed that the only predictor for malignant ventricular arrhythmia in NSTEMI patients treated with early invasive strategy was WBC [29]. A correlation between inflammation and ventricular arrhythmia in post MI patients is suspected, and post-mortem analysis of patients with sudden cardiac death sustains myocardial inflammation as a risk factor for ventricular arrhythmias [30]. WBC also correlated with heart failure occurrence in ACS and in NSTEMI patients with an acceptable discriminative power in our study. The study of Barron et al. also found that elevated WBC is associated with increased incidence of heart failure in patients with MI [24].

There are some suggested mechanisms considered as being the potential basis for correlating high levels of WBC with complications and/or death. Development of resistance to thrombolytic therapy is the natural result of changes (i.e., the state of hypercoagulability, leukocytes, indirect cardiotoxicity through pro inflammatory ischemia or reperfusion lesions/injury, and enlargement of the size of the infarct) in the microcirculation. Between all these factors, after an acute MI, leucocytes have a main function in the initial inflammatory response with a repairing role, which favors the formation of a scar (instead of necrotic tissue). According to experimental data that suggest the existence of a linear association between the size of necrotic tissue and the levels of leukocyte and systemic response, it can be deduced that the larger the size of the infarction, the greater the leukocyte response. It is obvious that infarcts of larger sizes more frequently predispose to the development of complications (i.e., heart failure, death); therefore, the fact that the size of the infarction is reflected in the number of red blood cells has a potential prognostic relevance [31].

NLR is the combination of two independent markers of inflammation and was found to be a predictor of short and long-term mortality in patients with ACS [6]. In several prospective studies NLR was found to be a useful marker to predict subsequent mortality in patients with ACS, STEMI [32] and NSTEMI [33] and also in patients who undergo PCI [34,35]. In our study NLR had ability to discriminate the risk of mortality, and a value over 5.116 had a 48.28% sensitivity and 81.23% specificity. In the present study admission NLR correlated with complication occurrence in ACS patients. Also, NLR was correlated with an increased risk of ventricular rhythm disturbances, especially in patients undergoing PCI. A retrospective study performed by Chaterjee et al. on a large number of patients who underwent angiography made the same observation that elevated NLR, WBC, and NEU were predictors of significant ventricular arrhythmias in patients with PCI [36]. NLR was an excellent factor for identification of increased risk of recurrent ischemic events in our study in patients with ACS and also in the subgroup of patients treated with interventional revascularization. Similar results were found in a prospective study on STEMI patients treated with PCI that observed that NLR was independently associated with no reflow phenomenon and in-hospital major cardiac events [37]. However, NLR was found to be a predictor of cardiovascular events in patients undergoing coronarography or cardiac revascularization in a meta-analysis performed by Wang et al. [38].

In a retrospective study performed by Ayca et al. a high NLR was associated with stent thrombosis in patients with STEMI [38]. A NLR >4.9 had 70% sensitivity and 65% specificity in predicting in-hospital mortality in STEMI patients [39]. Also, in our study NLR was significantly higher in STEMI patients and in stented patients that developed complications, but this parameter failed to identify complication risk. NLR was a predictor of heart failure in ACS patients in the present study. This is in concordance with the results observed by Tamhane et al. that described an association between NLR and heart failure [35].

LYM cause a modulation of the inflammatory response and a low LYM count is associated with more extensive atherosclerosis [32] and with a poor prognosis in patients with acute MI [6]. In our study, a lower LYM level predicted increased in-hospital complications in patients with UA, respectively MI development but the number of patients was very low. A lower LYM count was also associated with more extensive and severe coronary atherosclerosis. Other studies observed similar results, a low LYM level was a predictor of recurrent instability and death in patients with unstable angina in the study performed by Zouridakis et al. and is also associated with atherosclerosis progression [40].

A lower value of HGB is a predictor of in-hospital mortality and complications in ACS patients in our study. Also, a lower HGB is correlated with adverse outcome in STEMI patients and was found to be a predictor of ventricular rhythm disturbances and heart failure in ACS patients. Furthermore, a low HGB level at admission was correlated with recurrent myocardial ischemic events in PCI patients. In the INTERACT trial low HGB at admission was associated with recurrent ischemia [40,41]. Anemia is a powerful and independent predictor of adverse outcome in patients across the spectrum of ACS in many studies [42,43]. A low HGB level is a predictor for the development of major cardiac events in patients with ACS. In VALIDATE-SWEEDHEART study anemia in ACS patients undergoing PCI constitutes a risk factor for ischemic events and mortality [44].

PLR is a parameter which is describing the systemic inflammatory response and was correlated with in-hospital mortality in patients with UA from our study, but the number of these patients was low. Other studies also observed that PLR correlates with greater overall mortality in patients with NSTEMI [45] and STEMI [46]. PLR was found to be a predictor of short term recurrent ischemic events in our study in patients with UA and enables to predict recurrent myocardial ischemic in patients treated with PCI. Furthermore, PLR was correlated with atrial fibrillation occurrence in patients who underwent PCI. However, there is growing evidence for the role of inflammation in development and persistence of atrial fibrillation [47]. In a meta-analysis performed by Dong et al. in STEMI patients, it was observed that a high PLR predicts poor in-hospital and long-term prognosis in patients undergoing PCI [48]. Several retrospective and prospective studies in patients with STEMI found that a high PLR was a predictor of no-reflow phenomenon in patients undergoing PCI [49,50]. Sun at al. in a prospective study on STEMI patients observed that higher PLR is correlated with recurrent MI, heart failure and ischemic stroke [46]. Also, in our study, an increased value of PLR was a good predictor for heart failure in NSTEMI patients.

PDW indicates varied size of platelets. The presence of large sized immature platelets in patients with ACS is due to increased bone marrow activity. PDW was found to be a good predictor for long term mortality after AMI [50,51]. In our study we did not find a correlation between PDW and mortality, but we evaluated in-hospital and not long-term mortality. Also, an increased PDW correlated with severity of coronary artery disease in patients with ACS in a retrospective study performed by Bekler et al. [52]. In our study, no correlation was found between PDW and the severity and extent of coronary artery disease and similar results were observed in a prospective study performed by Luca et al. [53]. PDW correlates with the occurrence of heart failure in patients with ACS after PCI in the study of Kowara et al. [54] and also our study PDW was found to be a predictor for in-hospital heart failure development in ACS patients.

MPV is an indicator of platelet size and an activation marker and is correlated in several studies with adverse cardiac outcomes in patients with ACS. MPV is a predictor of impaired reperfusion and six-month mortality in patients with STEMI [55] and NSTEMI [56] who underwent PCI. On the contrary, other studies have suggested that there is no association between increased MPV and mortality in patients with coronary artery disease [57,58]. Similar results were found in our study. We did not find correlation between MPV and mortality or adverse outcome on short term in the present study. MPV could be influenced by previous medication consisting of statin therapy as described in other studies [59,60,61] or EDTA can increase platelet size by causing platelet swelling.

The mechanisms involved in understanding MPV are not yet fully known. In the case of patients with ACS, several theories explain the increased platelet volume, one of them stating that after consuming the normal platelets in size, several large, immature platelets are released into the bloodstream [62]. Another theory considers that in the case of few patients, the increased platelet volume is generated by the existence of default large platelets. Taking in account all the other hematological parameters, platelet count and MPV are usually inheritable among them [63].

Also, for patients who have suffered acute coronary events, the pathophysiology that underlies the increased levels of hematological markers mentioned and discussed above is still not clearly established. Literature data suggested specific mechanisms regarding certain parameters (e.g., MPV, WBC, and RDW); however, none of them enjoyed a global agreement in thei regard. Certainly, in order to optimize the treatment and management in patients with established coronary heart disease, additional research is needed to facilitate the understanding and explanation of the basic mechanism by which coagulation profile, hematological indices, ACS incidence and, as well, apparition and development of complications are associated [63,64].

In the present study the coagulation parameter PT was able to predict mortality and complications in ACS patients, including in the subgroup of patients interventional managed. PT had the best discriminative power for mortality with a value over 14.95 s having a sensitivity of 64.8% and a specificity of 80.1% in detecting patients with risk of death, especially in the subgroup of patients with STEMI. In another study in patients with ACS undergoing PCI, a prolonged PT measured at admission was associated with all-cause mortality and may be used to identify high risk patients [9]. Prothrombin time is a marker of coagulation abnormalities that was observed to be correlated with poor outcomes in critically ill patients in the study of Fuhrmann et al. [64] and with high mortality in the intensive care unit in the study of Fei et al. [65]. In patients who undergo coronary artery by-pass a prolonged PT was associated with increased mortality in the study of Hannan et al. [66]. In the progression of atherosclerosis coagulation and inflammation processes are involved. Due to activation of the coagulation process and to increased inflammation, consumptive coagulopathy may appear. Prolonged PT is related to inflammation which contributes to consumptive coagulopathy but can also result from severe comorbidities of the patients since coagulation factors are synthesized in the liver (hepatic dysfunction, heart failure).

Hematological parameters derived from hemogram obtained with modern automated equipment, wide and easily available in clinical practice, and also at an affordable price could become important tools for in-hospital clinical surveillance and for risk stratification of patients with ACS. These parameters add independent information to other clinical and paraclinical variables in short term risk stratification. According to recent studies, it seems that their prognostic value is increasing if they are used in combined analysis with other biomarkers or with risk scores like GRACE or SYNTAX score.

## 5. Conclusions

Both coagulation and hematological parameters are considered as having a good potential in being used as diagnostic and/or prognostic markers; they are also wide and easily available and can be correlated with short term in hospital outcome in patients with ACS. WBC, RDW, and PT were found to be predictors of poor outcome and can contribute to risk stratification of ACS patients and identification of those subjects that will need close monitoring and intensified treatment. The fastest and most efficient classification of those who are at higher risk is a valuable tool that facilitates their monitoring and careful management by the cardiologist; it also allows judicious and optimal programming in order to follow the patient’s evolution, thereby implicitly leading to the reduction of mortality.

## Figures and Tables

**Table 1:** Clinical/paraclinical data of the mortality group vs. discharged group in ACS patients.

Data	Clinical/Paraclinical Characteristics	Discharged (*n* = 849)	Mortality (*n* = 87)	*p*
Demographics	Age (Years)	64.90 ± 11.56	72.98 ± 11.60	<0.001
	Male	559/849 (65.84%)	42/87 (48.27%)	<0.001
	BMI (kg/m²)	30.07 ± 5.33	29.12 ± 4.59	0.06
Previous History	Smoking	201/849 (23.67%)	23/87 (26.44%)	0.232
	Diabetes Mellitus	261/849 (30.74%)	33/87 (37.93%)	0.172
	Hypertension	595/849 (70.08%)	30/87 (34.48%)	<0.001
	Dyslipidemia	360/849 (42.40%)	64/87 (73.56%)	<0.001
Diagnosis	UA	438/849 (51.59%)	3/87 (3.45%)	<0.001
	NSTEMI	101/849 (11.89%)	13/87 (14.94%)	0.409
	STEMI	310/849 (36.51%)	71/87 (81.61%)	<0.001
Number of Diseased Vessels	1	236/692 (34.10%)	5/28 (17.86%)	<0.001
	2	177/692 (25.58%)	1/28 (3.57%)	<0.001
	3	207/692 (29.91%)	21/28 (75%)	<0.001
CBC	HGB (g/dL)	13.78 ± 1.79	12.63 ± 2.19	<0.001
	WBC (×10³/μL)	10.45 ± 4.11	14.45 ± 6.90	0.006
	NEU (×10³/μL)	7.40 ± 3.84	11.19 ± 6.50	0.003
	LYM (×10³/μL)	2.16 ± 1.01	2.19 ± 1.59	0.391
	PLT (×10³/μL)	225.68 ± 70.91	237.84 ± 91.36	0.993
	PDW (%)	20.12 ± 1.24	19.95 ± 1.41	0.417
	MPV (fl)	8.39 ± 1.91	7.74 ± 1.52	0.124
	NLR	3.52 ± 4.08	6.45 ± 6.79	0.013
	PLR	128.17 ± 82.60	169.56 ± 117.23	0.010
	RDW (%)	12.39 ± 1.48	13.07 ± 1.26	0.001
Coagulation Tests	APTT(s)	31.82 ± 17.00	36.49 ± 16.77	0.096
	PT(s)	15.36 ± 6.69	17.85 ± 1.01	<0.001
Creatinine (mg/dL)	-	1.04 ± 0.56	1.72 ± 1.07	<0.001
LVEF (%)		47.09 ± 8.63	34,4 ± 9.87	<0.001

Legend: BMI—body mass index; UA—unstable angina; NSTEMI—non ST elevation myocardial infarction; STEMI—ST elevation myocardial infarction; CBC—complete blood count; HGB—hemoglobin; WBC—white blood cells; NEU—neutrophils; LYM—lymphocytes; PLT—platelets; PDW—platelet distribution width; MPV—mean platelet volume; NLR—neutrophils to lymphocyte ratio; PLR—platelet to lymphocyte ratio; RDW—red cell distribution width; APTT—activated partial thromboplastin time; PT—prothrombin time; LVEF—left ventricular ejection fraction.

**Table 2:** Hematological and coagulation parameters of the mortality group vs. discharged group in NSTEMI patients.

NSTEMI CBC	Discharged (*n* = 101)	Mortality (*n* = 13)	*p*
HGB (g/dL)	13.43 ± 1.87	12.36 ± 1.82	0.054
WBC (×10³/μL)	10.57 ± 4.196	17.00 ± 7.80	<0.001
NEU (×10³/μL)	7.60 ± 3.94	12.72 ± 7.75	0.001
LYM (×10³/μL)	2.09 ± 0.95	3.10 ± 2.33	0.06
PLT (×10³/μL)	222.88 ± 63.35	235.90 ± 78.76	0.49
PDW (%)	20.08 ± 0.99	20.03 ± 1.30	0.71
MPV (fl)	8.11 ± 1.53	7.50 ± 1.54	0.26
NLR	3.66 ± 5.22	5.29 ± 6.32	0.23
PLR	139.24 ± 110.94	134.19 ± 99.78	0.78
RDW (%)	12.47 ± 1.43	13.50 ± 1.11	0.01
APTT (s)	34.44 ± 14.48	36.56 ± 15.38	0.36
PT (s)	16.13 ± 6.39	17.76 ± 3.24	0.42

Legend: NSTEMI—non-ST elevation myocardial infarction; CBC—complete blood count; HGB—hemoglobin; WBC—white blood cells; NEU—neutrophils; LYM—lymphocytes; PLT—platelets; PDW—platelet distribution width; MPV—mean platelet volume; NLR—neutrophils to lymphocyte ratio; PLR—platelet to lymphocyte ratio; RDW—red cell distribution width; APTT—activated partial thromboplastin time; PT—prothrombin time.

**Table 3:** Hematological and coagulation parameters of the mortality group vs. discharged group in STEMI patients.

STEMI-CBC	Discharged (*n* = 310)	Mortality (*n* = 71)	*p*
HGB	14.10 ± 1.852	12.66 ± 2.29	0.017
WBC	12.36 ± 4.47	14.26 ± 6.72	0.006
NEU	9.19 ± 4.21	11.15 ± 6.32	0.003
LYM	2.21 ± 1.18	2.06 ± 1.43	0.130
PLT	241.94 ± 74.47	242.03 ± 92.33	0.099
PDW	20.12 ± 1.19	19.97 ± 1.44	0.655
MPV	8.18 ± 1.86	7.84 ± 1.52	0.133
NLR	5.16 ± 4.52	6.84 ± 6.97	0.013
PLR	138.68 ± 95.04	175.48 ± 121.61	0.01
RDW	12.29 ± 1.53	13.01 ± 1.29	0.001
APTT	31.54 ± 19.73	36.56 ± 17.35	0.218
PT	14.16 ± 5.29	17.92 ± 6.26	0.001

Legend: STEMI—ST elevation myocardial infarction; CBC—complete blood count; HGB—hemoglobin; WBC—white blood cells; NEU—neutrophils; LYM—lymphocytes; PLT—platelets; PDW—platelet distribution width; MPV—mean platelet volume; NLR—neutrophils to lymphocyte ratio; PLR—platelet to lymphocyte ratio; RDW—red cell distribution width; APTT—activated partial thromboplastin time; PT—prothrombin time.

**Table 4:** Clinical/paraclinical data of the groups with/without in-hospital complications in ACS patients.

Data	Clinical/Paraclinical Parameters	No Complications (*n* = 743)	Complications (*n* = 193)	*p*
Demographics	Age (Years)	64.51 ± 11.66	70.03 ± 11.28	<0.001
	Male	488/743 (65.68%)	113/193 (58.55%)	0.085
	BMI (kg/m²)	31.15 ± 5.38	29.90 ± 4.82	0.002
Previous history	Smoking	190/743 (25.57%)	34/193 (17.61%)	0.021
	Diabetes Mellitus	225/743 (30.28%)	69/193 (35.75%)	0.148
	Hypertension	524/743 (70.52%)	101/193 (52.33%)	<0.001
	Dyslipidemia	360/743 (48.45%)	64/193 (33.16%)	<0.001
Diagnosis	UA	39/743 (53.56%)	43/193 (22.28%)	<0.001
	NSTEMI	83/743 (11.17%)	30/193 (15.54%)	0.109
	STEMI	261/743 (35.13%)	120/193 (62.17%)	<0.001
Number of Diseased Vessels	1	198/605 (32.73%)	43/115 (37.39%)	0.146
	2	154/605 (25.45%)	24/115 (20.87%)	0.06
	3	184/605 (30.41%)	44/115 (38.26%)	0.003
CBC	HGB (g/dL)	13.84 ± 1.76	12.04 ± 2.09	<0.001
	WBC(×10³/μL)	10.18 ± 3.75	13.38 ± 6.38	<0.001
	NEU(×10³/μL)	7.16 ± 3.52	10.13 ± 5.98	<0.001
	LYM(×10³/μL)	2.15 ± 0.94	2.23 ± 1.49	0.37
	PLT (×10³/μL)	225.83 ± 71.45	230.56 ± 79.12	0.42
	PDW (%)	20.08 ± 1.21	20.15 ± 1.42	0.53
	MPV(fl)	8.37 ± 1.88	8.16 ± 1.88	0.21
	NLR	3.41 ± 3.94	5.30 ± 5.90	<0.001
	PLR	1267.21 ± 80.39	150.59 ± 107.62	0.013
	RDW (%)	12.31 ± 1.41	13.02 ± 1.56	<0.001
Coagulation Tests	APTT(s)	31.80 ± 17.49	34.20 ± 15.38	0.18
	PT(s)	15.20 ± 6,69	16.86 ± 6.27	0.018
Cholesterol (mg/dL)	-	177.34 ± 47.98	173.81 ± 45.72	0.34
Creatinine (mg/dL)	-	1.01 ± 0.51	1.45 ± 0.96	<0.001
LVEF%	-	47.74 ± 8.16	38.89 ± 10.90	<0.001

Legend: BMI—body mass index; UA—unstable angina; NSTEMI—non ST elevation myocardial infarction; STEMI—ST elevation myocardial infarction; CBC—complete blood count; HGB—hemoglobin; WBC—white blood cells; NEU—neutrophils; LYM—lymphocytes; PLT—platelets; PDW—platelet distribution width; MPV—mean platelet volume; NLR—neutrophils to lymphocyte ratio; PLR—platelet to lymphocyte ratio; RDW—red cell distribution width; APTT—activated partial thromboplastin time; PT—prothrombin time; LVEF—left ventricular ejection fraction.

**Table 5:** Hematological and coagulation parameters of the groups with/without in-hospital complications in NSTEMI patients.

NSTEMI CBC	No Complications (*n* = 84)	Complications (*n* = 30)	*p*
HGB (g/dL)	13.46 ± 1.82	12.88 ± 2.06	0.15
WBC (×10³/μL)	10.29 ± 3.77	14.93 ± 7.07	<0.001
NEU (×10³/μL)	7.39 ± 3.70	11.18 ± 6.53	0.002
LYM (×10³/μL)	2.09 ± 0.96	2.69 ± 1.82	0.059
PLT (×10³/μL)	223.44 ± 65.45	226.95 ± 64.83	0.80
PDW (%)	20.09 ± 0.99	19.87 ± 1.20	0.43
MPV (fl)	8.23 ± 1.51	7.44 ± 1.41	0.58
NLR	3.81 ± 5.45	4.26 ± 5.03	0.70
PLR	133.86 ± 104.45	149.69 ± 119.92	0.57
RDW (%)	12.24 ± 0.99	13.77 ± 1.88	<0.001
APTT (s)	31.95 ± 6.90	40.83 ± 21.24	0.03
PT (s)	15.50 ± 4.60	17.70 ± 7.46	0.21

Legend: NSTEMI—non-ST elevation myocardial infarction; CBC—complete blood count; HGB—hemoglobin; WBC—white blood cells; NEU—neutrophils; LYM—lymphocytes; PLT—platelets; PDW—platelet distribution width; MPV—mean platelet volume; NLR—neutrophils to lymphocyte ratio; PLR—platelet to lymphocyte ratio; RDW—red cell distribution width; APTT—activated partial thromboplastin time; PT—prothrombin time.

**Table 6:** Hematological and coagulation parameters of the groups with/without in-hospital complications in STEMI patients.

STEMI-CBC	No Complications (*n* = 261)	Complications (*n* = 120)	*p*
HGB (g/dL)	14.22 ± 1.78	12.98 ± 2.24	<0.001
WBC (×10 ³/μL)	12.00 ± 3.96	14.25 ± 6.49	<0.001
NEU (×10³/μL)	8.90 ± 3.72	10.97 ± 6.16	<0.001
LYM (×10³/μL)	2.17 ± 1.05	2.23 ± 1.57	0.67
PLT (×10³/μL)	243.29 ± 75.55	239.05 ± 83.24	0.62
PDW (%)	20.09 ± 1.14	20.10 ± 1.56	0.95
MPV (fl)	8.16 ± 1.83	8.05 ± 1.76	0.62
NLR	5.01 ± 4.36	6.47 ± 6.31	0.01
PLR	138.59 ± 93.81	159.37 ± 114.39	0.07
RDW (%)	12.21 ± 1.45	12.89 ± 1.56	<0.001
APTT (s)	32.00 ± 21.42	33.14 ± 14.07	0.61
PT (s)	14.23 ± 5.80	16.07 ± 5.09	0.01

Legend: STEMI—ST elevation myocardial infarction; CBC—complete blood count; HGB—hemoglobin; WBC—white blood cells; NEU—neutrophils; LYM—lymphocytes; PLT—platelets; PDW—platelet distribution width; MPV—mean platelet volume; NLR—neutrophils to lymphocyte ratio; PLR—platelet to lymphocyte ratio; RDW—red cell distribution width; APTT—activated partial thromboplastin time; PT—prothrombin time.

**Table 7:** Hematological and coagulation parameters of the groups with/without in-hospital complications in ACS patients treated with PCI.

PCI-CBC	No Complications (*n* = 404)	Complications (*n* = 86)	*p*
HGB (g/dL)	14.05 ± 1.68	13.59 ± 1.74	0.02
WBC (×10³/μL)	10.79 ± 3.82	12.60 ± 5.79	0.001
NEU (×10³/μL)	7.64 ± 3.58	9.22 ± 5.20	0.002
LYM (×10³/μL)	2.25 ± 0.97	2.49 ± 1.73	0.105
PLT (×10³/μL)	231.274 ± 73.43	231.44 ± 64.13	0.96
PDW (%)	20.12 ± 1.18	20.22 ± 1.33	0.55
NLR	3.55 ± 3.41	4.77 ± 5.62	0.01
PLR	120.39 ± 64.44	142.06 ± 118.14	0.03
MPV (fl)	8.38 ± 1.94	8.19 ± 1.81	0.42
RDW (%)	12.13 ± 1.10	12.65 ± 1.14	<0.001
APTT (s)	32.09 ± 21.76	34.02 ± 18.15	0.56
PT (s)	14.09 ± 4.80	16.36 ± 7.15	0.007

Legend: PCI—percutaneous coronary intervention; CBC—complete blood count; HGB—hemoglobin; WBC—white blood cells; NEU—neutrophils; LYM—lymphocytes; PLT—platelets; PDW—platelet distribution width; MPV—mean platelet volume; NLR—neutrophils to lymphocyte ratio; PLR—platelet to lymphocyte ratio; RDW—red cell distribution width; APTT—activated partial thromboplastin time; PT—prothrombin time.

**Table 8:** Hematological and coagulation parameters of the groups with/without ventricular rhythm disturbances in ACS patients.

ACS-CBC	No Ventricular Arrhythmias (*n* = 880)	Ventricular Arrhythmias (*n* = 56)	*p*
HGB (g/dL)	13.73 ± 1.820	12.79 ± 2.23	<0.001
WBC (×10³/μL)	10.49 ± 4.18	15.89 ± 6.95	<0.001
NEU (×10³/μL)	7.45 ± 3.92	12.57 ± 6.53	<0.001
LYM (×10³/μL)	2.15 ± 1.01	2.40 ± 1.75	0.11
PLT (×10³/μL)	226.09 ± 72.63	238.09 ± 79.72	0.23
PDW (%)	20.11 ± 1.26	19.99 ± 1.24	0.55
MPV (fl)	8.37 ± 1.88	7.60 ± 1.72	0.664
NLR	3.60 ± 4.18	7.01 ± 7.11	<0.001
PLR	130.04 ± 85.14	160.63 ± 109.29	0.01
RDW (%)	12.40 ± 1.42	13.27 ± 1.88	<0.001
APTT(s)	32.19 ± 16.78	35.15 ± 18.09	0.29
PT (s)	15.57 ± 6.72	16.63 ± 5.52	0.26

Legend: ACS—acute coronary syndrome CBC—complete blood count; HGB—hemoglobin; WBC—white blood cells; NEU—neutrophils; LYM—lymphocytes; PLT—platelets; PDW—platelet distribution width; MPV—mean platelet volume; NLR—neutrophils to lymphocyte ratio; PLR—platelet to lymphocyte ratio; RDW—red cell distribution width; APTT—activated partial thromboplastin time; PT—prothrombin time.

**Table 9:** Hematological and coagulation parameters of the groups with/without ventricular arrhythmias in STEMI patients.

STEMI-CBC	No Ventricular Arrhythmias (*n* = 332)	Ventricular Arrhythmias (*n* = 49)	*p*
HGB (g/dL)	13.99 ± 1.92	12.76 ± 2.35	0.074
WBC (×10³/μL)	12.27 ± 4.47	15.64 ± 6.97	<0.001
NEU (×10³/μL)	9.12 ± 4.22	12.43 ± 6.57	<0.001
LYM (×10³/μL)	2.17 ± 1.14	2.35 ± 1.72	0.364
PLT (×10³/μL)	242.99 ± 77.51	235.00 ± 81.54	0.862
PDW (%)	20.10 ± 1.23	20.06 ± 1.25	0.798
MPV (fl)	8.19 ± 1.81	7.67 ± 1.74	0.992
NLR	5.20 ± 4.66	7.30 ± 7.25	0.001
PLR	142.27 ± 98.94	162.79 ± 112.02	0.066
RDW (%)	12.29 ± 1.40	13.33 ± 1.94	0.458
APTT (s)	32.16 ± 20.07	33.68 ± 15.02	0.077
PT (s)	14.49 ± 5.58	16.78 ± 5.67	0.055

Legend: STEMI—ST elevation myocardial infarction; CBC—complete blood count; HGB—hemoglobin; WBC—white blood cells; NEU—neutrophils; LYM—lymphocytes; PLT—platelets; PDW—platelet distribution width; MPV—mean platelet volume; NLR—neutrophils to lymphocyte ratio; PLR—platelet to lymphocyte ratio; RDW—red cell distribution width; APTT—activated partial thromboplastin time; PT—prothrombin time.

**Table 10:** Hematological and coagulation parameters of the groups with/without ventricular rhythm disturbances in PCI treated patients.

PCI-CBC	No Ventricular Arrhythmias (*n* = 466)	Ventricular Arrhythmias (*n* = 24)	*p*
HGB (g/dL)	13.99 ± 1.71	13.58 ± 1.64	0.23
WBC (×10³/μL)	10.79 ± 3.81	16.54 ± 7.40	<0.001
NEU (×10³/μL)	7.64 ± 3.54	12.73 ± 6.78	<0.001
LYM (×10³/μL)	2.26 ± 1.08	2.88 ± 2.02	0.11
PLT(×10³/μL)	230.77 ± 72.60	241.64 ± 54.62	0.35
PDW (%)	20.14 ± 1.20	20.09 ± 1.37	0.83
MPV (fl)	8.38 ± 1.92	7.80 ± 1.79	0.15
NLR	3.63 ± 3.74	6.29 ± 5.91	0.001
PLR	123.22 ± 73.66	139.70 ± 118.53	0.32
RDW (%)	12.20 ± 1.13	12.62 ± 1.00	0.07
APTT (s)	32.12 ± 20.45	37.02 ± 25.77	0.33
PT (s)	14.55 ± 5.54	15.62 ± 5.09	0.41

Legend: PCI—percutaneous coronary intervention; CBC—complete blood count; HGB—hemoglobin; WBC—white blood cells; NEU—neutrophils; LYM—lymphocytes; PLT—platelets; PDW—platelet distribution width; MPV—mean platelet volume; NLR-neutrophils to lymphocyte ratio; PLR—platelet to lymphocyte ratio; RDW—red cell distribution width; APTT—activated partial thromboplastin time; PT—prothrombin time.

**Table 11:** Hematological and coagulation parameters between the groups with/without heart failure in ACS patients.

ACS-CBC	No Heart Failure (*n* = 820)	Heart Failure (*n* = 116)	*p*
HGB (g/dL)	13.77 ± 1.80	12.98 ± 2.11	0.006
WBC (×10³/μL)	10.48 ± 4.18	13.28 ± 6.35	<0.001
NEU (×10³/μL)	7.44 ± 3.91	9.99 ± 6.01	<0.001
LYM (×10³/μL)	2.16 ± 1.00	2.17 ± 1.46	0.981
PLT (×10³/μL)	225.49 ± 71.97	236.11 ± 80.22	0.207
PDW (%)	20.08 ± 1.22	20.21 ± 1.53	0.160
MPV (fl)	8.34 ± 1.87	8.21 ± 1.96	0.847
NLR	3.57 ± 4.32	5.39 ± 5.25	<0.001
PLR	128.39 ± 82.54	157.35 ± 111.39	<0.001
RDW (%)	12.36 ± 1.44	13.12 ± 1.49	<0.001
PT (s)	15.38 ± 6.67	17.08 ± 6.35	0.302
APTT (s)	32.10 ± 17.26	34.21 ± 15.38	0.33

Legend: ACS—acute coronary syndrome; CBC—complete blood count; HGB—hemoglobin; WBC—white blood cells; NEU—neutrophils; LYM—lymphocytes; PLT—platelets; PDW—platelet distribution width; MPV—mean platelet volume; NLR—neutrophils to lymphocyte ratio; PLR—platelet to lymphocyte ratio; RDW—red cell distribution width; APTT—activated partial thromboplastin time; PT—prothrombin time.

**Table 12:** Hematological and coagulation parameters of the groups with/without heart failure in NSTEMI patients.

NSTEMI CBC	No Heart Failure (*n* = 92)	Heart Failure (*n* = 22)	*p*
HGB (g/dL)	13.491 ± 1.769	12.563 ± 2.219	0.338
WBC (×10³/μL)	10.432 ± 3.912	15.476 ± 7.459	0.005
NEU (×10³/μL)	7.453 ± 3.713	11.785 ± 7.039	0.001
LYM (×10³/μL)	2.164 ± 1.091	2.564 ± 1.746	0.027
PLT (×10³/μL)	224.495 ± 64.075	223.818 ± 70.423	0.885
PDW (%)	20.105 ± 1.033	19.878 ± 1.012	0.886
MPV (fl)	8.210 ± 1.536	7.336 ± 1.317	0.276
NLR	3.601 ± 5.297	5.076 ± 5.495	0.111
PLR	131.574 ± 102.688	163.572 ± 130.222	0.034
RDW (%)	12.236 ± 0.984	14.158 ± 1.883	0.026
APTT (s)	34.148 ± 14.356	36.184 ± 3.597	0.317
PT (s)	15.553 ± 6.669	18.021 ± 7.966	0.231

Legend: NSTEMI—non-ST elevation myocardial infarction; CBC—complete blood count; HGB—hemoglobin; WBC—white blood cells; NEU—neutrophils; LYM—lymphocytes; PLT—platelets; PDW—platelet distribution width; MPV—mean platelet volume; NLR—neutrophils to lymphocyte ratio; PLR—platelet to lymphocyte ratio; RDW—red cell distribution width; APTT—activated partial thromboplastin time; PT—prothrombin time.

**Table 13:** Hematological and coagulation parameters of the groups with/without heart failure in STEMI patients.

STEMI-CBC	No Heart Failure (*n* = 303)	Heart Failure (*n* = 78)	*p*
HGB (g/dL)	14.00 ± 1.96	13.16 ± 2.12	0.152
WBC (×10³/μL)	12.44 ± 4.62	13.69 ± 6.13	0.001
NEU (×10³/μL)	9.31 ± 4.33	10.45 ± 5.91	0.001
LYM (×10³/μL)	2.20 ± 1.16	2.13 ± 1.48	0.083
PLT (×10³/μL)	241.34 ± 78.08	244.36 ± 78.01	0.932
PDW (%)	20.09 ± 1.16	20.12 ± 1.52	0.297
MPV (fl)	8.12 ± 1.83	8.15 ± 1.71	0.564
NLR	5.29 ± 5.01	6.17 ± 5.42	0.027
PLR	140.22 ± 96.90	164.11 ± 114.12	0.024
RDW (%)	12.29 ± 1.40	12.84 ± 1.31	0.333
APTT (s)	31.90 ± 20.19	34.25 ± 15.98	0.539
PT (s)	14.51 ± 5.84	15.98 ± 4.61	0.305

Legend: STEMI—ST elevation myocardial infarction; CBC—complete blood count; HGB—hemoglobin; WBC—white blood cells; NEU—neutrophils; LYM—lymphocytes; PLT—platelets; PDW—platelet distribution width; MPV—mean platelet volume; NLR—neutrophils to lymphocyte ratio; PLR—platelet to lymphocyte ratio; RDW—red cell distribution width; APTT—activated partial thromboplastin time; PT—prothrombin time.

**Table 14:** Hematological and coagulation parameters of the groups with/without heart failure in PCI treated patients.

PCI-CBC	No Heart Failure (*n* = 448)	Heart Failure (*n* = 42)	*p*
HGB (g/dL)	14.00 ± 1.70	13.64 ± 1.74	0.18
WBC (×10³/μL)	11.03 ± 4.22	11.83 ± 4.76	0.26
NEU (×10³/μL)	7.84 ± 3.91	8.59 ± 4.22	0.26
LYM (×10³/μL)	2.29 ± 1.06	2.33 ± 1.71	0.83
PLT (×10³/μL)	231.74 ± 72.36	226.62 ± 66.46	0.63
PDW (%)	20.10 ± 1.18	20.49 ± 1.37	0.09
MPV (fl)	8.34 ± 1.91	8.43 ± 2.02	0.79
NLR	3.64 ± 3.77	5.05 ± 4.99	0.02
PLR	121.54 ± 69.04	149.09 ± 127.84	0.03
RDW (%)	12.16 ± 1.09	12.86 ± 1.25	<0.001
APTT (s)	32.47 ± 21.38	33.01 ± 17.77	0.88
PT (s)	14.36 ± 5.06	16.54 ± 7.90	0.04

Legend: PCI—percutaneous coronary intervention; CBC—complete blood count; HGB—hemoglobin; WBC—white blood cells; NEU—neutrophils; LYM—lymphocytes; PLT—platelets; PDW—platelet distribution width; MPV—mean platelet volume; NLR—neutrophils to lymphocyte ratio; PLR—platelet to lymphocyte ratio; RDW—red cell distribution width; APTT—activated partial thromboplastin time; PT—prothrombin time.

**Table 15:** Hematological and coagulation parameters of the groups with/without re infarction in ACS patients treated with PCI.

PCI-CBC	No Re Infarction (*n* = 486)	Re Infarction (*n* = 4)	*p*
HGB (g/dL)	13.97 ± 1.70	13.74 ± 1.00	0.67
WBC (×10³/μL)	11.10 ± 4.28	10.66 ± 3.28	0.83
NEU (×10³/μL)	7.90 ± 3.94	9.09 ± 3.46	0.60
LYM (×10³/μL)	2.29 ± 1.13	0.94 ± 0.44	0.03
PLT (×10³/μL)	231.36 ± 71.96	224.00 ± 59.23	0.82
PDW (%)	20.142 ± 1.207	19.64 ± 0.327	0.10
MPV (fl)	8.35 ± 1.92	7.49 ± 0.33	0.12
NLR	3.71 ± 3.79	10.13 ± 10.76	0.001
PLR	123.41 ± 76.05	217.58 ± 115.22	0.03
RDW (%)	12.22 ± 1.13	12.77 ± 0.75	0.32
APTT (s)	32.56 ± 20.98	27.80 ± 15.02	0.82
PT (s)	14.52 ± 5.29	38.6 ± 6.55	<0.001

Legend: ACS—acute coronary syndrome; PCI—percutaneous coronary intervention; CBC—complete blood count; HGB—hemoglobin; WBC—white blood cells; NEU—neutrophils; LYM—lymphocytes; PLT—platelets; PDW—platelet distribution width; MPV—mean platelet volume; NLR—neutrophils to lymphocyte ratio; PLR—platelet to lymphocyte ratio; RDW—red cell distribution width; APTT—activated partial thromboplastin time; PT—prothrombin time.

## Data Availability

All data are available in the archive (data base) of the Clinical County Emergency Hospital of Oradea, Oradea, Bihor County, Romania.
